# Attenuation of spatial bias with target template variation

**DOI:** 10.1038/s41598-024-57255-z

**Published:** 2024-04-03

**Authors:** Injae Hong, Min-Shik Kim

**Affiliations:** 1 Visual Attention Lab, Brigham and Women’s Hospital, Boston, MA 02215 USA; 2 Harvard Medical School, Boston, MA 02115 USA; 3 Department of Psychology, Yonsei University, Seoul, 03722 Republic of Korea

**Keywords:** Target template, Location probability learning, Spatial bias, Visual search, Psychophysics, Human behaviour

## Abstract

This study investigated the impact of target template variation or consistency on attentional bias in location probability learning. Participants conducted a visual search task to find a heterogeneous shape among a homogeneous set of distractors. The target and distractor shapes were either fixed throughout the experiment (target-consistent group) or unpredictably varied on each trial (target-variant group). The target was often presented in one possible search region, unbeknownst to the participants. When the target template was consistent throughout the biased visual search, spatial attention was persistently biased toward the frequent target location. However, when the target template was inconsistent and varied during the biased search, the spatial bias was attenuated so that attention was less prioritized to a frequent target location. The results suggest that the alternative use of target templates may interfere with the emergence of a persistent spatial bias. The regularity-based spatial bias depends on the number of attentional shifts to the frequent target location, but also on search-relevant contexts.

## Introduction

Visual search is ubiquitous in human life, from everyday events (e.g., Where’s Waldo? Is there a typo in this article?) to professional settings (e.g., Does this bag contain a prohibited item for the airport? If so, where and what is it?). In a variety of visual search situations, people are often asked to find a target whose specific features are not defined. For example, a prohibited item may be a “gun” or it may be a “knife”. The security officer should search for a target even though the exact shape of such an item is not defined. Similarly, a fast and accurate visual search is required even if the specific target template does not exist.

Target template has been widely discussed in the visual search literature. To name just a few, top–down knowledge of the target biases covert and overt attention towards the template-matching target^1–3^ or toward a target-defining feature^4^. The more specific the target template is, the more effective it is for visual search. For example, perceptually matching target to target template tends to bias attention more than perceptually mismatching target or schematic/semantic information^5^. A target template facilitates attentional guidance and aids target decision-making by increasing the efficiency of template-to-target comparison^6,7^.

A target template can be fixed and specific on each trial, but it can sometimes be variant and unpredictable. When the target template is not fixed^7^ or exists only as a schema or category^8^, attentional guidance can be impaired. It is questionable whether a variant and unpredictable target template would affect biased visual search, as it does under standard search circumstances. Imagine a situation where you usually go to your bedroom first to find your phone because you have learned from your experience that you often leave your phone on the bed. However, if your target is sometimes a phone, sometimes a blanket, and sometimes a pair of glasses, all of which are often found in your bedroom, will you go to your bedroom first to search for any of the possible targets?

Bias in visual search can result from accumulated experiences of finding the target in a particular location many times, similar to the habit of going to your bedroom first. If a target frequently appears in a specific area of the visual display, individuals can learn this statistical regularity and prioritize their spatial attention toward the most frequent target location^9–12^. Location probability learning is a form of *location*-based learning and is independent of target features^13–15^. Given the nature of the attentional bias induced by location probability learning, changing the target template during a biased search is unlikely to affect spatial bias. Yet, there remains the possibility that spatial bias may not be as strong or persistent when the target template is not fixed and unpredictable. Regularity-based spatial bias is known to base distinct attentional mechanism from explicit and goal-driven attention^16–18^, and top-down attention may interact with such bias when required^14^.

The current study aimed to investigate the influence of consistent and varying target templates on the persistence or size of spatial bias. The search items were hexagons and octagons, and the task was to find a singleton shape among a homogeneous set of distractors. A target-consistent group only looked for a hexagon (or octagon) among octagons (or hexagons), and the target-distractor shapes remained unchanged. In contrast, a target-variant group looked for any singleton shape among different-shaped distractors. This task was revised from Bacon and Egeth^19^ which suggested the influence of attentional control settings on visual search against a salient distractor. Note that the current study prevents any possible influence of bottom-up attention, as the items in a search array shared the identical color. Furthermore, this type of manipulation, in which target templates are mixed up or fixed during visual search, has been found to be a reliable paradigm for understanding the role of target unpredictability on distractor suppression processing^20,21^. In addition to the manipulation on target templates, the target was presented in one of the quadrants with a higher probability than the other quadrants. We hypothesized that the degree of spatial bias would decrease or disappear if variable target templates were introduced, disrupting the probability learning of target location. The magnitude of spatial bias was compared in the scenarios with varying and consistent target templates.

## Experiment 1

### Method

#### Participants

A total of 48 participants took part in the experiment in exchange for monetary compensation or course credits. The sample size was determined based on Wang and Theeuwes^22^ and Jiang et al.^13^, which predicted 13 or 12 participants per each group with an expected power of 0.90 and *α* error probability of 0.05. We doubled the sample size to account for possible dropouts after the awareness check (see “Results”). All participants confirmed having normal or corrected-to-normal visual acuity and provided informed consent prior to the experiment. The study was approved by institutional review board of Yonsei University and was performed in accordance with the relevant guidelines and regulations.

#### Apparatus and stimuli

**Figure 1:**
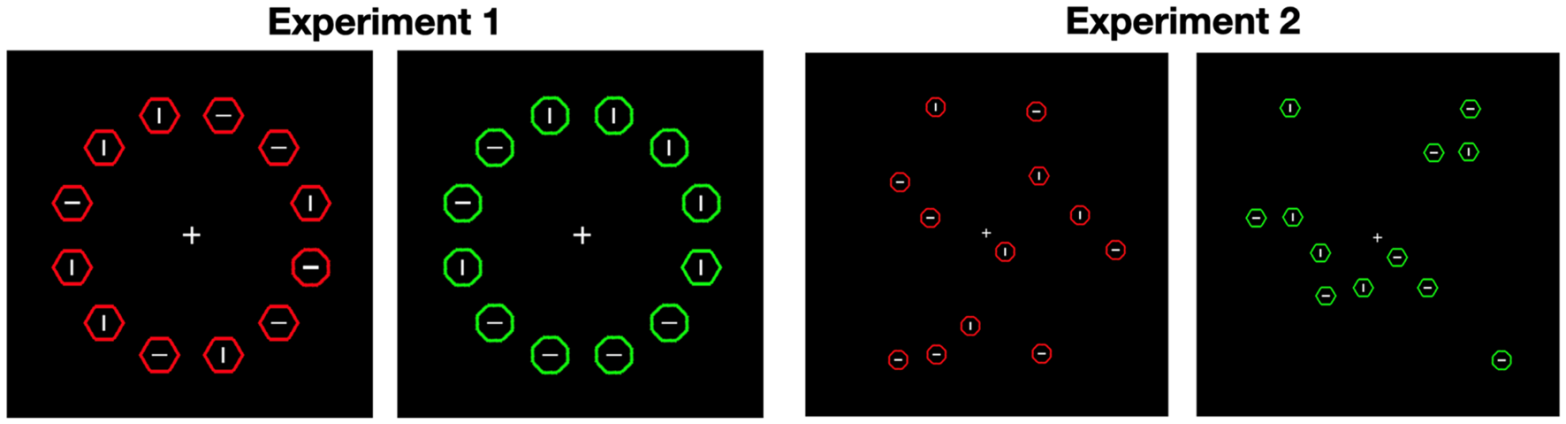
Sample displays of Experiments 1 and 2.

Search stimuli were displayed on a black background (RGB: 0, 0, 0). The stimuli consisted of twelve items equidistantly arranged on an imaginary circle with a radius of 4.3°. Search items were either eleven hexagons and one octagon, or eleven octagons and one hexagon. The size of each item subtended 1.4° × 1.4°. Search items were colored either in red (RGB: 255, 0, 0) or green (RGB: 0, 255, 0), and 12 items had the same color at any given time. Each search item contained a vertical or horizontal bar with a long side of 0.6° and a short side of 0.1°. These bars were all colored white (RGB: 255, 255, 255). The sample displays are depicted in Fig. 1.

#### Procedure

The search task comprised of 32 practice trials and 480 experimental trials, divided into ten blocks with 48 trials each. The first eight blocks constituted the training phase, while the last two blocks constituted the testing phase. Participants took a self-paced break between blocks.

Each trial began with a 500 ms white fixation at the center of the display. Search items were displayed at predetermined locations. The task was to find a shape singleton among homogeneous shape sets and to report whether the bar in the shape singleton was vertical or horizontal. Participants pressed ‘z’ if the bar was vertical and ‘/’ if the bar was horizontal. The response time was limited to 4 s. The screen went blank as soon as the response was made or when the response window ended, with a random interval between 500 and 750 ms before the next trial began. If participants responded incorrectly or did not respond within the response window, an error message appeared for 1 s before the next trial.

Participants were assigned either to the target-variant or target-consistent group. In the target-variant group, the target shape was not defined. The target could be either a hexagon or an octagon. In half of the trials, the target item was a hexagon, and the non-target items were octagons. In the other half of the trials, the target item was an octagon, and the non-target items were hexagons. Two different target types were intermixed. Therefore, the target shape was not predicted until the stimulus onset. For the target-consistent group, the target shape was cued before the search task began. Half of the participants in the target-consistent group were assigned to find a hexagon among 11 octagons, while the remaining participants were instructed to find an octagon among 11 hexagons. The color of the search item was randomly chosen between red and green, and the color was not predictive of the target location or shape.

Item locations were defined according to a statistical regularity. The search display was divided into invisible quadrants, and each quadrant contained three search items. In 50% of the total trials, a target appeared in one of four quadrants (‘rich’ quadrant). In the remaining 50% of trials, the target was equally likely to appear in one of other three quadrants (‘sparse’ quadrants). This probability amounts to 16.67% of the trials in each of the three quadrants. A specific target location was randomly selected from three possible locations in a target-containing quadrant. The remaining locations were filled with non-target distractors. Quadrant-based target probability was applied only during the training phase. During the testing phase, the target appeared in each quadrant with equal probability, 25% per quadrant. Although the target was presented equally often in all four quadrants, the previously rich quadrant was still called the rich quadrant and the previously sparse quadrant was called the sparse quadrant. The rich quadrant was consistent throughout the task, but was counterbalanced across participants.

The search tasks were followed by an awareness check session. All participants were asked whether they noticed that the singleton shape appeared with high probability in a certain quadrant. All participants were forced to answer either ‘yes’ or ‘no’. Then, regardless of their answers, participants ranked the quadrants in order of the expected probability of a shape singleton appearing in them.

### Results

The data sets were primarily screened on the basis of the response to the awareness check. Six participants who responded that they had noticed that the target often appeared in a particular location and ranked the rich quadrant as the one in which the shape singleton was most likely to appear were removed. Those participants are expected to have little or no spatial bias, irrespective of target template variation, because attentional guidance by explicit goal is transient^16^.

Trials with response not being made within the 4 s response window were excluded, representing 3.04% of the total trials The total accuracy, after excluding one participant whose accuracy was lower than 70%, was 94.84% (*SD* = 4.58%). The accuracy of the target-consistent group was 95.9% (*SD* = 3.19%) and the accuracy of the target-variant group was 93.7% (*SD* = 5.66%). Incorrect trials were not included in the reaction time (RT) analysis.

We tested whether the extent of spatial bias is affected by an unpredictable target template. RTs of the training and testing phases were individually analyzed using a generalized linear mixed effect model (GLMM) with the *afex* package^23^. This analysis allows for a trial-based analysis while controlling for individual differences and possible random effects. The fixed effects were the target location (rich, sparse) as a within-subject factor and the target variability (target-consistent, target-variant) as a between-subject factor. The random effect was the by-subject intercepts. RT was fitted with the inverse gamma function. Significance tests against the null model were performed with the likelihood ratio test^24^. The Bonferroni method was used to adjust the *p*-value adjustment of the follow-up analysis.

For the training phase, the fixed effect of the target location was significant, *χ*^*2*^(1) = 682.982, *p* < 0.001, a faster RT in the rich quadrant than in the sparse quadrants. That is, spatial attention was spatially biased toward the target’s frequent location, facilitating target detection when it appeared in the rich quadrant than in the sparse quadrants. The fixed effect of the target variability was also significant, *χ*^*2*^(1) = 12.608, *p* < 0.001. RT was faster in the target-consistent group than in the target-variant group, and the RT difference may be due to search difficulty or impaired attentional guidance^7^ due to varying target templates. Both the target-consistent and target-variant groups showed a significant spatial bias toward the rich quadrant, target-consistent group *z* = 27.47,* p* < 0.001; target-variant group *z* = 8.537, *p* < 0.001. However, the extent of bias from the target-consistent group was significantly larger than that from the target-variant group, *χ*^*2*^(1) = 226.387, *p* < 0.001. This means that participants with variant target templates tended to prioritize the frequent target quadrant less than those with fixed target templates, even when they found the target equally often in the rich quadrant.

Does the extent and persistence of spatial bias differ depending on target template variation? RT from the testing phase was compared to test whether the extent and persistence of spatial bias differed by target template variation. Remember that target was presented equally often in every quadrant. Hence, an RT difference between previously rich and previously sparse quadrants without statistical regularity would indicate the persistence of spatial bias.

**Figure 2:**
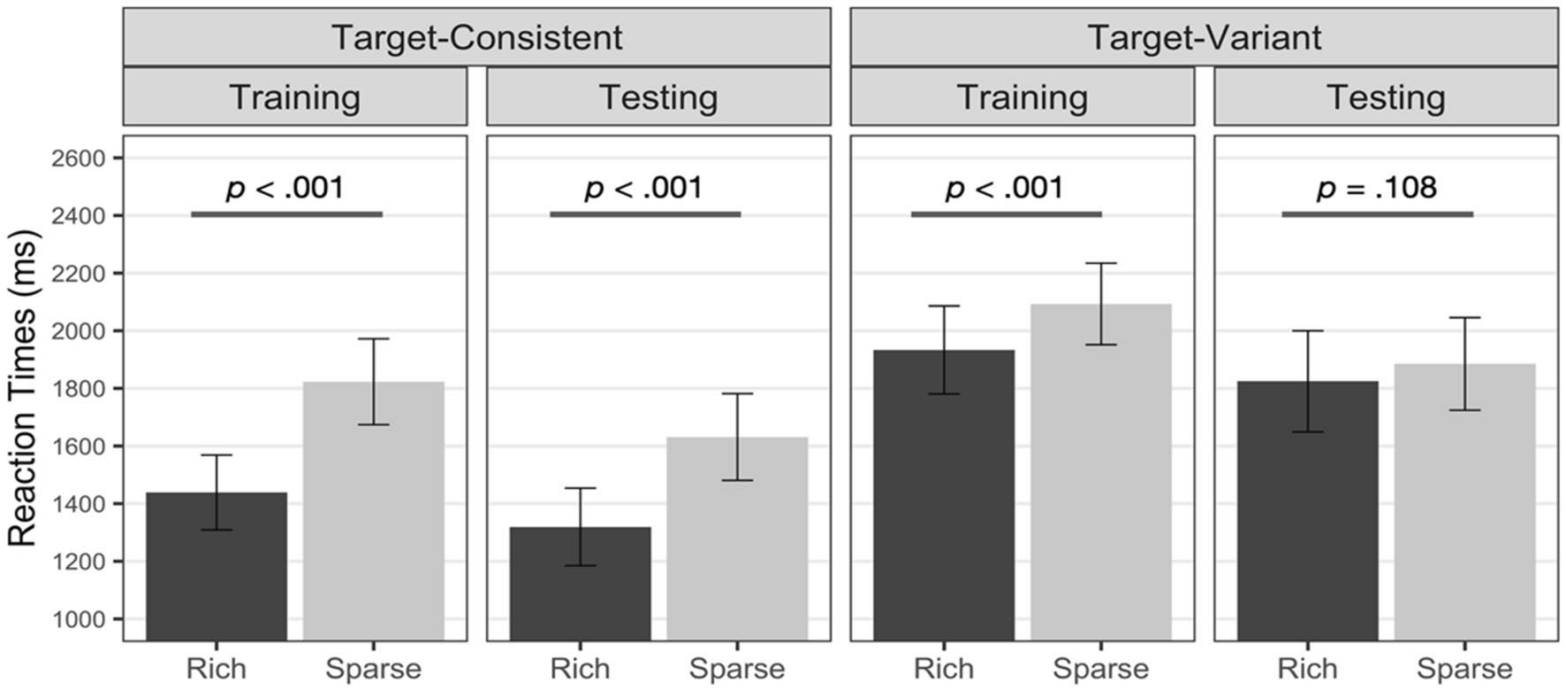
Mean RT by phase of the target-consistent and target-variant groups of Experiment 1. Error bars represent 95% confidence intervals.

During the testing phase, detecting the target was quicker in the target-consistent group compared to the target-variant group, *χ*^*2*^(1) = 9.941, *p* = 0.002, and the target detection was faster in the rich quadrant than the sparse quadrants, *χ*^*2*^(1) = 93.418, *p* < 0.001. Importantly, target variability by target’s location interaction was significant, *χ*^*2*^(1) = 55.763, *p* < 0.001. While the target-consistent group maintained consistent spatial bias until the testing phase, *z* = 10.93, *p* < 0.001, the target-variant group failed to maintain location probability learning effect, *z* = 1.606, *p* = 0.108. Mean RTs are plotted in Fig. 2.

### Discussion

Experiment 1 examined how inconsistency of target templates affected biased visual search. When the target template remained consistent throughout the task, spatial attention was persistently guided to the frequent target location. However, when the target template varied during training, spatial bias was not persistent until the statistical rule was eliminated. Changes in the target template caused participants to fail maintaining long-term statistical knowledge.

Overall detection time was slower in the target-variant group than in the target-consistent group. The longer RT when the target templates changed suggests a possible explanation that task difficulty, not the target template variation per se, interfered with emergence of long-term bias. However, this argument is unlikely: If the task difficulty were solely responsible for influencing spatial bias, one might expect the target-variant group, where the target was harder to discriminate, to demonstrate a pronounced spatial bias as a compensatory mechanism to enhance search efficiency. Contrary to this expectation, spatial bias was not markedly sustained in the target-variant group across phases, indicating that factors beyond mere task difficulty contribute to the observed patterns of spatial bias. Also, scientific evidence supports that the size of spatial bias was persistent despite the changes in search efficiency^13^, and learning of multiple regularities was also available irrespective of task difficulty^14^.

## Experiment 2

Experiment 1 demonstrated that when searching for targets with variant and inconsistent target templates, spatial bias is less stable and persistent. The number of attentional shifts toward a frequent target location was identical between target-consistent and target-variant conditions, but only the target-consistent group showed a persistent spatial bias.

It is questionable whether the emergence of a spatial bias is completely prevented by unpredictive target templates or occurs at an attenuated level. In Experiment 1, the search items were placed at equidistant locations on an imaginary circle, and the locations were fixed across trials. Because target detection is relatively easier on a well-arranged display, attenuation of the spatial bias by a variant target template could nullify its existence. By increasing the size of search displays and decreasing the location specificity from Experiment 1, we increased the distance between attentional shifts, enhancing the benefit of spatial bias and sensitivity of RT for this measure. We expected participants to rely on location probability in visual search, if target variation did not completely prevent the emergence of spatial bias. We tested whether spatial bias persists, but is reduced, when target templates are unpredictable during biased search.

### Method

#### Participants

A total of 50 participants participated in exchange for cash or course credits. Two participants were dropped due to a technical error. Among 48 participants, 24 participants were assigned to the target-consistent group, and the remaining 24 participants were assigned to the target-variant group.

#### Apparatus and stimuli

Stimuli and apparatus were identical to Experiment 1 except for the search array. The 18.89° × 20.17° search display was divided into 8 × 8 matrices, and 64 cells were equally assigned to four invisible quadrants. Three specific cells out of 16 in a quadrant were randomly selected, and the search items were located in the center of the selected cells, jittered ± 0.27° vertically and horizontally. The sample displays are depicted in Fig. 1.

#### Procedure

The procedure of Experiment 2 was identical to Experiment 1.

### Results

The data were preprocessed and analyzed using the same methods as in Experiment 1. First, three participants were excluded based on the report of the awareness check sessions. The number of excluded trials whose RTs did not reach the 4 s response window was 5.91%. Then, three participants were removed from the target-variant group whose accuracy did not reach 70%. The total accuracy of the remaining data was 93.0% (*SD* = 5.34%). The accuracy of the target-consistent group was 94.7% (*SD* = 4.59%) and the accuracy of the target-variant group was 91.1% (*SD* = 5.58%). The target-variant group’s accuracy was numerically lower because the task was more difficult, and participants often failed to meet the response window.

RT of the training phase by target’s location (rich, sparse) and target consistency (target-consistent, target-variant) were submitted to GLMM. In the training phase, the general RT was faster in the target-consistent group than in the target-variant group, *χ*^*2*^(1) = 11.783, *p* < 0.001, and the target detection was faster in the rich quadrant than in the sparse quadrants, *χ*^*2*^(1) = 573.604, *p* < 0.001. The interaction between target’s location and target consistency was significant, *χ*^*2*^(1) = 149.897, *p* < 0.001. Follow-up analysis showed that both the target-consistent and target-variant groups showed an attentional bias toward the rich quadrant, target-consistent group: *z* = 24.51, *p* < 0.001; target-variant group: *z* = 8.807, *p* < 0.001. The significant interaction implies that the target-consistent group showed a greater spatial bias than the target-variant group.

**Figure 3:**
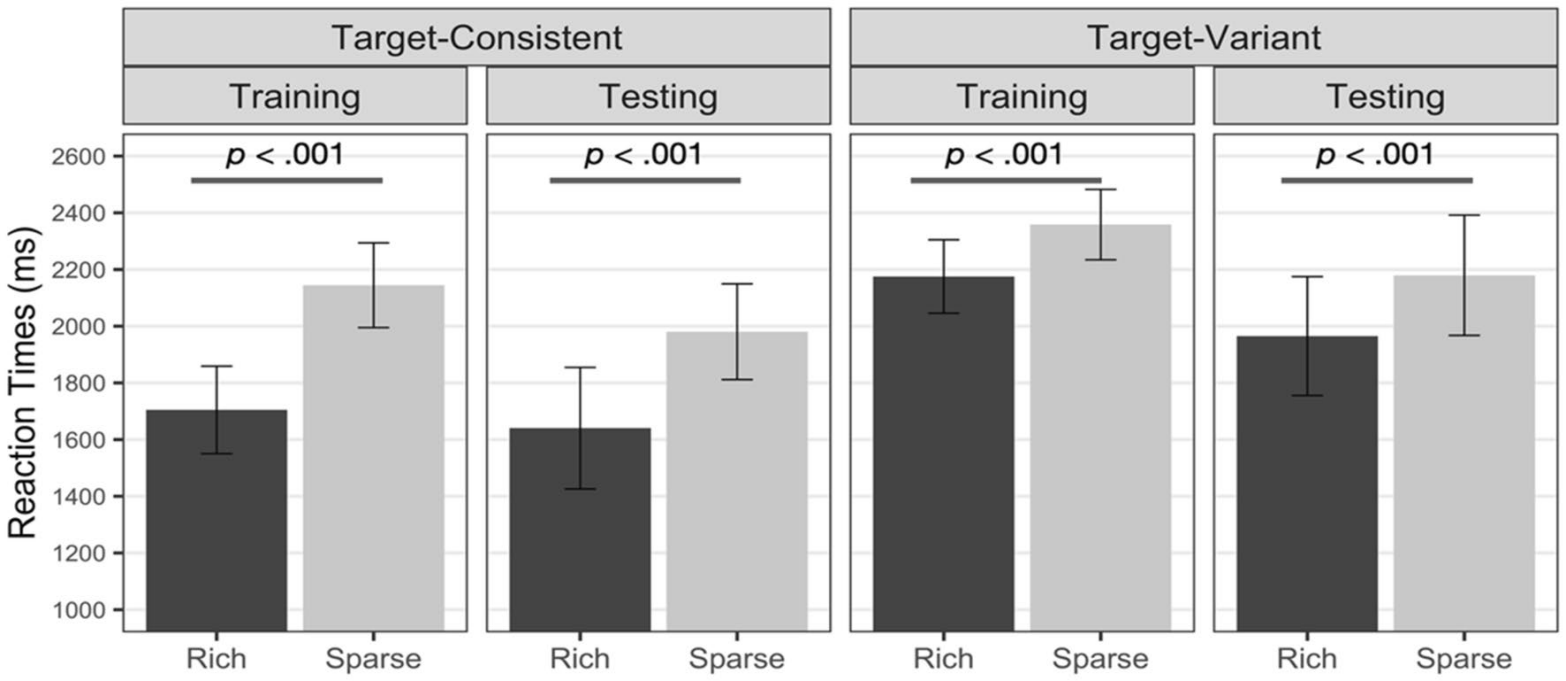
Mean RT by phase of the target-consistent and target-variant groups of Experiment 2. Error bars indicate 95% confidence intervals.

RT of the testing phase were analyzed to determine whether the persistence of spatial bias after removing statistical regularity would differ by target consistency. As a result, RT was not significantly different by target consistency, *χ*^*2*^(1) = 1.131, *p* = 0.288, and the rich quadrant still gained attentional priority over the sparse quadrants, *χ*^*2*^(1) = 76.980, *p* < 0.001. Importantly, the interaction between the target location and the target consistency was statistically significant, *χ*^*2*^(1) = 11.173, *p* < 0.001. As in the training phase, both the target-consistent and the target-variant groups showed a significant attentional bias toward the rich quadrant, target-consistent group: *z* = 8.00, *p* < 0.001; target-variant group: *z* = 3.908, *p* < 0.001. Although target variability did not completely prevent attentional learning, constant changes in target templates during learning did reduce the extent of bias. Mean RT is depicted in Fig. 3.

### Discussion

Experiment 2 examined the influence of target template variation on spatial bias when visual search is more demanding with increased need for spatial bias. As a result, a significant attentional bias toward the rich quadrant was observed in both the target-consistent and target-variant groups. Notably, spatial bias was comparatively reduced in the target-variant group compared to the target-consistent group.

## General discussion

This study investigated whether constantly changing the target template could interfere with biased visual search. When the target template was randomly varied during visual search with location-related rule, the magnitude of spatial bias was reduced compared to when the target template was invariant. This suggests that regularity-based spatial biases can be affected by dynamic search environments in which target features are not constant.

Active alteration of target templates during visual search affects the emergence of spatial bias, but it does not affect after the emergence of spatial bias^25^. While the present study showed an interference effect of featural inconsistency on spatial bias, previous studies have shown resistance of existing bias despite target template changes (i.e., item features, search difficulty^13^). Spatial bias seems to be influenced by the search context, but once bias is learned, it is inflexible and robust^12^.

What contributed to the attenuated spatial bias when the target template was unpredictive? One possible explanation is the strategic reallocation of attentional resources, influenced by the search strategy. With the target explicitly defined by its shape, participants might have prioritized the shape dimension, thereby diminishing the resources available for learning about location probability. Conversely, in scenarios where the target template remained constant, participants were more likely able to allocate a greater amount of their attentional resources towards location-based learning, thus enhancing their spatial bias. Another possibility is the concurrent executive load imposed when alternating between target templates. The target-variant group should have maintained twice as many target templates and kept switched between two of them, compared to the target-variant group. Concurrent working memory maintenance load may slow down visual search, but does not impair learning of location probability^26^. However, it is unknown whether the concurrent use of executive function impairs the emergence of long-term spatial bias. The acquisition of a useful strategy for visual search, spatial bias, may have been delayed as search efficiency was impaired by executive load^27,28^. Further studies are needed to understand the relationship between executive load and location probability learning.

Although the magnitude of the spatial bias was attenuated, this study confirmed that the spatial bias can persist despite variations in the target template. This suggests that the emergence of attentional bias is regularity-based, occurs on a spatial map and is (at least partially) independent of target features^14,29^. However, regularity-based spatial bias does not imply complete ignorance of featural dynamics or target object shapes. Featural dynamics affect spatial bias in a way that it reduces the extent of learning.

In summary, this study investigated the role of variant target templates in the emergence and persistence of spatial bias during visual search. The target template was either kept constant or was varied during visual search with location-based statistical regularity. As a result, spatial bias was somewhat reduced when the target template was varied. Target template variability did not binarily affect spatial bias, suggesting that biased search is available to some extent when the search environment requires more attention. Statistical learning allows for spatial bias, but the extent of bias can be flexibly adapted to the varying search contexts.

## Data Availability

The datasets generated and analyzed during the current study are available in the Open Science Framework repository, https://osf.io/x6c8z/?view_only=44d8f92b28c547de99cb1417ddaae3f1.
